# MicroRNA-26a regulates glucose metabolism by direct targeting PDHX in colorectal cancer cells

**DOI:** 10.1186/1471-2407-14-443

**Published:** 2014-06-16

**Authors:** Bing Chen, Yuling Liu, Xuewen Jin, Weiliang Lu, Jingjing Liu, Zijing Xia, Qiong Yuan, Xia Zhao, Ningzhi Xu, Shufang Liang

**Affiliations:** 1 State Key Laboratory of Biotherapy/Collaborative Innovation Center for Biotherapy, West China Hospital, Sichuan University, No.17, the third section of Renmin South Road, Chengdu 610041, P. R. China; 2 Department of Pharmacology, Medical College, Wuhan University of Science and Technology, Wuhan 430065, P. R. China; 3 Gynecologic and Pediatric Diseases and Birth Defects of Ministry of Education, West China Second Hospital, Sichuan University, Chengdu 610041, P. R. China; 4 Laboratory of Cell and Molecular Biology, Cancer Institute and Cancer Hospital, Chinese Academy of Sciences, Beijing 100034, P. R. China

**Keywords:** MicroRNA-26a, PDHX, Colorectal cancer, Glucose metabolism

## Abstract

**Background:**

Reprogramming energy metabolism has been an emerging hallmark of cancer cells. MicroRNAs play important roles in glucose metabolism.

**Methods:**

The targets of microRNA-26a (miR-26a) were predicted by bioinformatics tools. The efficacy of miR-26a binding the 3′-untranslated region (UTR) of pyruvate dehydrogenase protein X component (PDHX) mRNA was evaluated using a dual-luciferase reporter assay. The PDHX expression at the mRNA and protein level in several colon cancer cell lines was quantified with real-time PCR and Western blot analysis respectively. The effects of miR-26a on glucose metabolism were determined by detecting the content of glucose consumption, production of lactate, pyruvate, and acetyl-coenzyme A.

**Results:**

The expression of miR-26a is inversely associated with the level of its targeting protein PDHX in several colon cancer cell lines with different malignancy potentials. MiR-26a inhibits PDHX expression by direct targeting the 3′-UTR of PDHX mRNA. The glucose consumption and lactate concentration were both greatly increased in colon cancer cells than the normal colon mucosal epithelia under physiological conditions. The overexpression of miR-26a in HCT116 cells efficiently improved the accumulation of pyruvate and decreased the production of acetyl coenzyme A. Meanwhile the inhibition of miR-26a expression induced inverse biological effects.

**Conclusions:**

MiR-26a regulates glucose metabolism of colorectal cancer cells by direct targeting the PDHX, which inhibits the conversion of pyruvate to acetyl coenzyme A in the citric acid cycle.

## Background

Recent studies have shown that microRNAs (miRNAs) play important roles in energy metabolism [1], and thus in cancer cells the alterations of cellular metabolism are associated with miRNA dysregulation [2,3]. MiRNAs participate in cell metabolism by regulating the expression of genes whose protein products either directly regulate metabolic machinery or indirectly modulate the expression of metabolic enzymes, serving as master regulators [4]. For example, microRNA-195-5p [5] and microRNA-143 [6] can repress the glucose uptake and glycolysis processing by inhibiting the expression of glucose transporter 3 (GLUT3) and hexokinase 2 respectively. And microRNA-143 could affect the glucose metabolism by regulating the AKT signaling pathway [7].

Reprogramming energy metabolism has been an emerging hallmark of cancer [8]. The best characterized metabolic phenotype in cancer cells is the “Warburg effect”. Even in the presence of oxygen, cancer cells can reprogram their glucose metabolism, and thus the ATP generation shifts from oxidative phosphorylation to glycolysis, leading to a state termed “aerobic glycolysis” [9]. Unlike normal cells, cancer cells usually derive a substantial amount of energy from aerobic glycolysis by converting most incoming glucose to lactate rather than metabolizing it through oxidative phosphorylation. Although ATP production via glycolysis can be more rapid than by oxidative phosphorylation, the ATP generated is far less for per unit of glucose consumed. As a result, this shift demands that cancer cells implement an abnormally high rate of glucose uptake to meet their increased energy and biosynthesis. By now, several studies have focused on miRNA contributions to the “Warburg effect” [1,3], and many molecules regulated by miRNAs are investigated.

MicroRNA-26a (miR-26a) shows a higher expression level in colon cancer tissues than in normal colon tissues [10], and its expression is also elevated under hypoxia conditions [11]. Meanwhile, miR-26a has been reported to play functions in cellular differentiation, cell growth, cell apoptosis and metastasis [12-15]. However, none of these studies on miRNA-26a focuses on energy metabolism. Several glycolysis genes, including glyceraldehyde-3-phosphate dehydrogenase, phosphoglycerate kinase 1 and phosphoglycerate mutase 1, have up-regulation in colon cancer tissues [16], therefore colon cancer cells have a higher glycolytic rate compared with normal cells [17]. In this study, we aim to discover the function of miR-26a on glucose metabolism in colorectal cancer cells. We have identified the pyruvate dehydrogenase protein X component (PDHX) as a direct target of miR-26a that is involved in the biological processes of glucose metabolism in colorectal cancer (CRC) cells.

## Methods

### Cell culture

The CRC cell lines including SW620, SW480 and HCT116 were ordered from American Type Culture Collection. The cell line NCM460, which is derived from normal human colon mucosal epithelium, was ordered from the INCELL Corporation, LLC, USA (http://www.incell.com). The cells were all cultured in Dulbecco’s modified Eagle’s medium (DMEM) supplemented with 10% fetal bovine serum at 37°C in humidified atmosphere with 5% (v/v) CO_2_.

### Prediction of candidate miRNA targets

The alignment of the miR-26a seed region and 3′-untranslated regions (UTR) of PDHX mRNA of different species were analyzed as literatures described previously [6,18]. The possible targets of miR-26a were screened by bioinformatics tools, Pictar (http://pictar.mdc-berlin.de) and TargetScan (http://www.targetscan.org).

### Construction of expression plasmids

A 461-bp nucleotide fragments in pre-miR-26a sequences (Additional file 1: Table S1) were cloned into the pENTR-CMV-EGFP vector (ordered from Wuhan Cell marker Biotechnology Co., Ltd., Wuhan, P. R. China) between *XhoI* and *EcoRI* to construct miR-26a expression plasmid, pENTR-miR-26a. The empty vector pENTR-MIRNA was used as a control in the ectopic overexpression of miR-26a.

**Figure 1:**
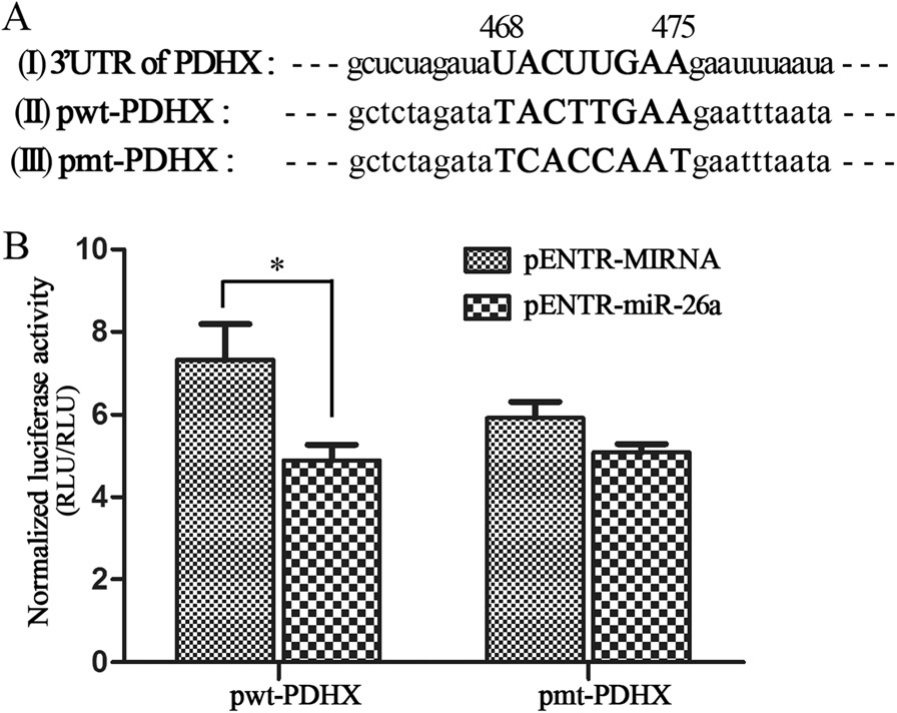
**MiR-26a targets the 3′UTR of PDHX mRNA directly. A**(I) The miR-26a matches the eight nucleotide sequences (468-475 nt, UACUUGAA) of the 3′ UTR of the PDHX mRNA; **A**(II) The 3′UTR of PDHX mRNA was amplified and the cDNA fragment was cloned to construct the wild type recombinant plasmid pwt-PDHX, which contains the eight nucleotide sequences (…TACTTGAA…); **A**(III) The relevant eight nucleotides (…TACTTGAA…) were mutated to a random sequence (…TCACCAAT…) to construct the mutant recombinant plasmid pmt-PDHX. **B**. The miR-26a targets the 3′ UTR of PDHX mRNA analyzed by the luciferase reporter assays. Both of the two luciferase signals were measured and the activity of the *Renilla* luciferase was normalized to the firefly luciferase to generate the normalized *Renilla* luciferase activity. In the case of pwt-PDHX (left), the expression of miR-26a reduced luciferase activity effectively, while the luciferase activity was not inhibited in the case of the pmt-PDHX (right). Data are shown as the mean ± the standard error of the mean (SEM) of three replicates. P-value was computed using the Student’s *t*-test. *p < 0.05. pwt-PDHX: wild type recombinant plasmid of 3′UTR of PDHX mRNA; pmt-PDHX: mutant recombinant plasmid of 3′UTR of PDHX mRNA; pENTR-miR-26a: the miR-26a expression plasmid; pENTR-MIRNA: the empty vector without miR-26a.

The 3′-untranslated region (3′UTR) of PDHX mRNA (Additional file 2: Table S2) was amplified by RT-PCR. The cDNA fragment corresponding to the 3′UTR of PDHX mRNA was cloned in the downstream of the *Renilla* luciferase gene in the psiCHECK-2 vector (Cat. # C8021, Promega, USA), which contains a reporter gene *Renilla* luciferase and an intraplasmid transfection normalization gene, a firefly luciferase. The 3′UTR of PDHX mRNA contains eight nucleotides (5′…UACUUGAA…3′), which are corresponding to miR-26a seed sequences (3′…AUGAACUU 5′) (Figure 1A(I)). In the wild type recombinant plasmid pwt-PDHX, the relevant eight nucleotides (…TACTTGAA…) were involved (Figure 1A(II)). Meanwhile, in the mutant recombinant plasmid pmt-PDHX (Figure 1A(III)), the eight nucleotides were mutated into a random nucleotide sequence (…TCACCAAT…).

### Cells treated with MiR-26a inhibitor

The miR-26a inhibitor was commercially available chemically modified antisense oligonucleotide inhibitor (Product ID: MH10249, Cat. #4464084, Life Technologies Corporation, USA). The non-targeting oligonucleotides (Cat. #4464076, Life Technologies Corporation, USA), as a negative control, were also ordered from this company.

The miR-26a inhibitor or the negative control was respectively transfected at a working concentration of 30 nM using Lipofectamine 2000 reagent (Cat. #11668-019, Invitrogen). The ratio of Lipofectamine 2000 reagent (μL) versus the oligonucleotides (pmol) was 18:1.

### RNA extraction and real-time PCR

Total RNA was extracted using Trizol reagent (Cat. #15596-026, Invitrogen). The total RNA was dissolved with RNase-free water for use. The first-strand cDNA for miR-26a was generated using a cDNA synthesis kit (Cat. #K1622, Thermo Scientific). Specific primers were designed for the reverse transcription of miR-26a and U6. The primer sequences for miR-26a were TGTCAACGATACGCTACCTAACGGCATGACAGTGTCAGCCTA. And the primer for U6 was GAACGCTTCACGAATTTGC based on literature reports [13]. For PDHX, random primers were used for mRNA reverse transcription (Cat. #170-8890, Bio-Rad).

The real-time PCR was performed to measure the relative expression using the Supermix-Bio-Rad kit (Cat. #172-5261, Bio-Rad) following the protocol. The U6 was used as an internal reference to calculate the relative expression level of miRNA, and the GAPDH mRNA level was taken as a comparison base for the target RNA expression. The relative RNA expression was calculated with the comparative CT method, which was normalized to the internal references. The forward RT-PCR primer for PDHX was 5′-GTCCCTCTAAAGCAGCTCAAAA-3′, and the reverse one was 5′-CTCCCTTCAAAAGATCCAACTG-3′. The forward one for miR-26a was 5′-CTGTCAACGATACGCTAC-3′, and its reverse was 5′-GTAATCCAGGATAGGCTG-3′. In addition, the forward RT-PCR primer for U6 was 5′-CTTCGGCAGCACATATAC-3′, meanwhile its reverse primer was 5′-GAACGCTTCACGAATTTGC-3′.

### Cell transfection

The miR-26a expression plasmid pENTR-miR-26a or the empty vector pENTR-MIRNA was respectively transfected at a working concentration of 100 nM using Lipofectamine 2000 reagent (Cat. #11668-019, Invitrogen). The ratio of DNA (μg) versus the Lipofectamine 2000 reagent (μL) was 1: 2.

For the promoter luciferase reporter assay, the plasmid pwt-PDHX or pmt-PDHX was co-transfected with the miR-26a expression vector pENTR-miR-26a or the empty vector pENTR-MIRNA into HEK293T cells. As mentioned above, Lipofectamine 2000 was used as the transfection reagent. The plasmid was used at a working concentration of 100 nM as well. The ratio of plasmid DNA (μg) versus the Lipofectamine 2000 reagent (μL) was 1: 2.

### Western blot analysis

The protein PDHX was detected against its specific antibody by western blot. The PVDF membranes were respectively incubated with the primary antibody of PDHX (diluted 1:1000, Cat. # S0394, Epitomics, USA) at 4°C overnight, followed by incubating with a secondary HRP-conjugated antibody at 37°C for 1 h. Signal detection was performed with Luminata Crescendo Western HRP Substrate (Cat. # WBLUR0100, Millipore). The detection of GAPDH using its antibody (Cat. # sc-365062, Santa Cruz Biotechnology) was taken as a control.

### Luciferase reporter assay

Either the plasmid pwt-PDHX or pmt-PDHX was respectively co-transfected with the miR-26a expression vector pENTR-miR-26a into HEK293T cells, and cells were harvested for assessment of luciferase activity at 48 hours after transfection. The luciferase activities of cellular extracts were measured with a Dual Luciferase Reporter Assay System (Cat. #E1910, Promega). Both *Renilla* luciferase and firefly luciferase activities were measured. The luciferase signal was normalized to the firefly luciferase signal as described previously [19].

### Measurement of glucose consumption and lactate production

Either the pENTR-miR-26a or miR-26a inhibitor was transfected into CRC cells. Cell culture media were collected after transfection for 48 h. Glucose uptake and lactate production were measured using Amplex^®^ Red Glucose/Glucose Oxidase Assay Kit (Cat. #A22189; Invitrogen) and lactate assay kit (Cat. #MAK064; Sigma-Aldrich) respectively. The results were normalized on the basis of total cellular protein amounts.

### Pyruvate assay

The concentration of pyruvate in CRC cells, transfected with pENTR-miR-26a or miR-26a inhibitor, was respectively measured using pyruvate assay kit (Cat. #K609-100; BioVision). Briefly, cells were collected after transfection for 48 h and dissolved with 0.5 ml of pyruvate assay buffer. And 50 μl sample was added with 50 μl of reaction mixture to incubate at room temperature for 30 minutes. A standard curve covering a range of 10–0.1 nmol per well was used as control. Absorbance was measured at 570 nm. The pyruvate concentration, which was normalized on the basis of totally cellular protein amounts, was calculated relative to the standard curve.

### Acetyl-coenzyme aassay

To analyze the production of acetyl coenzyme A (acetyl-CoA), cell extracts were prepared using the perchloric acid approach as described previously, with minor modification [20]. CRC cells, treated with pENTR-miR-26a or miR-26a inhibitor, were harvested and washed with phosphate-buffered saline. Cells were mixed in 1 mL of washing buffer (10 mM sodium phosphate [pH 7.5], 10 mM MgCl_2_, 1 mM EDTA), treated with 200 μL of 3 M ice-cold HClO_4_, and incubated on ice for 30 minutes. The mixture was centrifuged for 5 minutes at 10,000 × g at 4°C. The supernatant was neutralized with saturated KHCO_3_ and centrifuged as described above. The level of acetyl-CoA in the cell extraction was quantified using the acetyl-CoA assay kit (Cat. # K317-100; BioVision). Fluorescence was measured (Ex/Em = 535/589 nm), and the acetyl-CoA concentration was calculated based on the standard curve. The production of acetyl-CoA was normalized based on the total of cellular proteins.

## Results

### Bioinformatics analysis for potential targets of miR-26a

**Figure 2:**
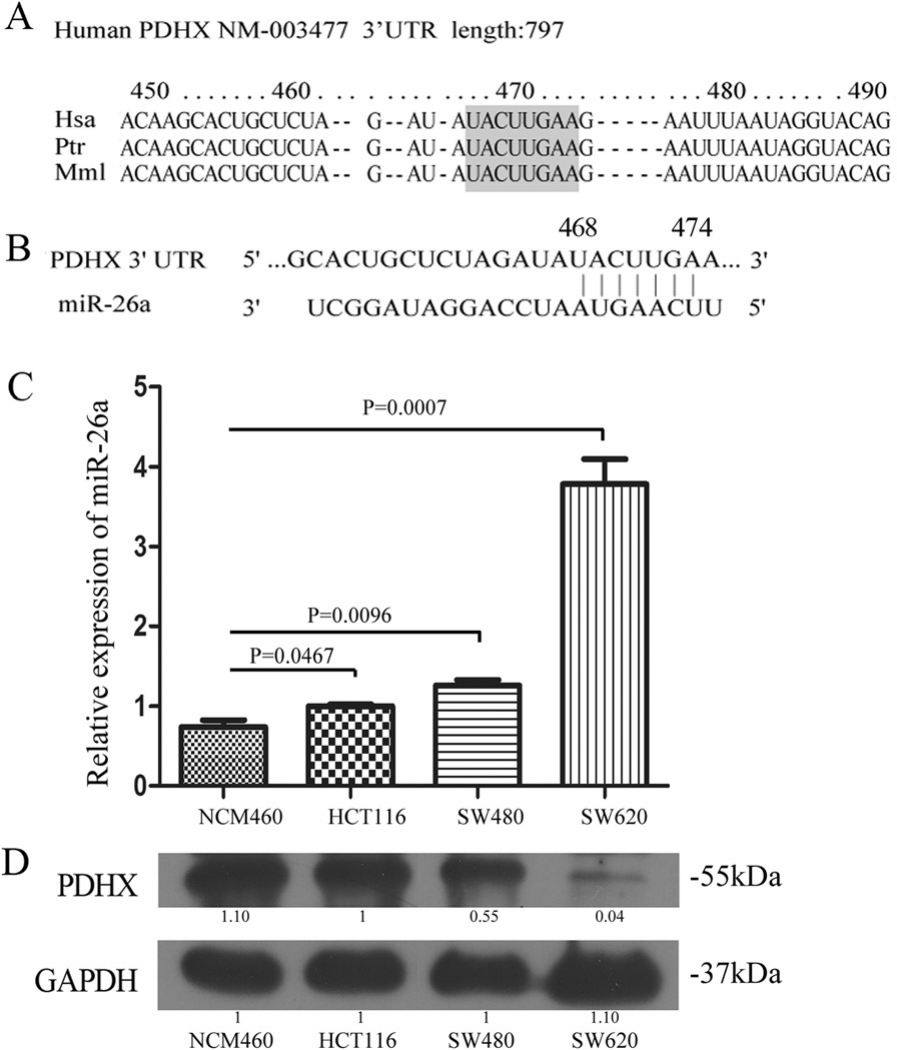
**PDHX is a predicted target of miR-26a. (A)** Alignment of the miR-26a seed region with 3′UTR of PDHX mRNA of different species across *Homo sapiens* (Hsa), *Pan troglodytes* (Ptr) and *Mus musculus* (Mmu). Bioinformatics analysis was performed using the TargetScan 6.2 software available at http://www.targetscan.org. The accession number of the protein PDHX from Has, Ptr and Mml was NM_003477, NM_001280073, NM_175094. The miR-26a seed region was highlighted in grey. **(B)** The 468–475 nt of PDHX 3′ UTR mRNA showed an exact match to the seed region of mature miR-26a. **(C-D)** MiR-26a level is inversely correlated with PDHX protein level in CRC cells. Expression of miR-26a and PDHX protein were respectively analyzed by qRT–PCR **(C)** and western blot **(D)**. The error bar represented the standard error of the mean (SEM). A Student’s *t*-test was performed to compare the differences between NCM460 and HCT116, SW480 or SW620.

By analyzing the 3′UTR mRNA of several glycolytic enzymes, we found that the 3′UTR of PDHX is relatively long with around 797 nucleotides, suggesting that PDHX mRNA is a possible target for miRNAs. In order to predict the possible target gene of miR-26a, the miRNA binding sites in the 3′UTR of PDHX were further analyzed through the Pictar and TargetScan software [6,18]. A conserved miR-26a binding site exists in the 3′UTR of PDHX across *Homo sapiens* (Hsa), *Pan troglodytes* (Ptr) and *Mus musculus* (Mmu) (Figure 2A), further we predicted an exact match to the seed region of mature miR-26a (Figure 2B). The bioinformatics analysis indicated a potential functional link between PDHX and miR-26a.

### MiR-26a level is inversely associated with the protein expression of PDHX

The expression level of miR-26a in NCM460, HCT116, SW480 and SW620 cells was examined using q-PCR analysis (Figure 2C). Compared with the normal epithelial cells NCM460, the expression of miR-26a was significantly increased to 1.3, 1.7 and 4.6-fold for the colon cancer cell lines HCT116, SW480 and SW620 respectively (p < 0.05). Generally, the expression level of miR-26a was significantly higher in malignant CRC cell lines HCT116, SW480 and SW620 than in a normal colorectal mucosal epithelial cell line NCM460 [21], especially it was almost increased over 4-fold in SW620 cells, which has high lymph node metastatic potentials [22]. Among the three CRC cell lines, the expression levels of miR-26a were gradually increased from HCT116 to SW480 and SW620 cells. Compared with HCT116 cells, which is derived from a human colorectal adenocarcinoma [22], SW480 and SW620 cells are typical model systems for CRC metastasis. The SW480 is derived from the primary site of CRC and SW620 comes from the recurrent lymph node metastasis [23]. Therefore, it has a positive correlation between the expression levels of miR-26a and the malignant degree of CRC cells.As miRNA expression is often inversely correlated with those of their specific target mRNAs, we further analyzed the protein expression of the miR-26a target —PDHX, in CRC cell lines. We found that the expression of PDHX was stepwise decreased in NCM460, HCT116, SW480 and SW620 cells (Figure 2D). The expression level of miR-26a in NCM460 was the lowest among the four cell lines (Figure 2C), but the protein level of PDHX exhibited the highest (Figure 2D). While in SW620 cells, the highest level of miR-26a expression and the lowest level of PDHX expression were observed. By comparing the expression levels of miR-26a and PDHX, an inverse correlation between them was observed in CRC cell lines, which supported the bioinformatics prediction on miR-26a target as above.

### PDHX is a direct target of miR-26a

**Figure 3:**
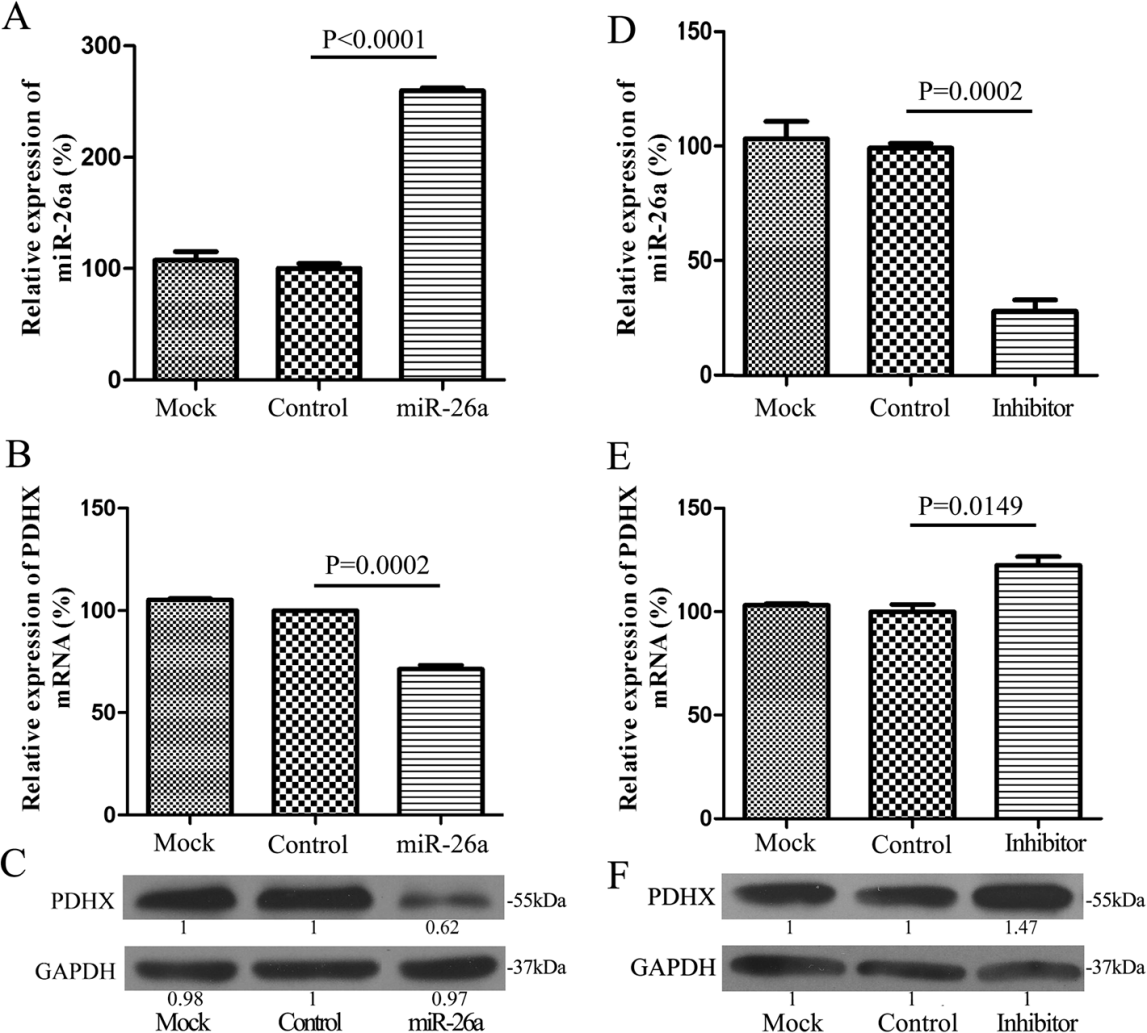
**A negative correlation between the expression level of miR-26a and PDHX in HCT116 cells.** The ectopic overexpression of miR-26a **(A)** induced a significant reduction of PDHX expression both in mRNA **(B)** and protein levels **(C)** in HCT116 cells. The empty plasmid pENTR-MIRNA was used as a control. While the expression of PDHX at both mRNA and protein levels **(E, F)** was increased in HCT116 cells along with an inhibition of miR-26a expression **(D)**. The non-targeting oligonucleotide was used as a control. The untreated HCT116 cells were taken as the mock object. The mean values were calculated from three separate experiments. SEM: the standard error of the mean.

**Figure 4:**
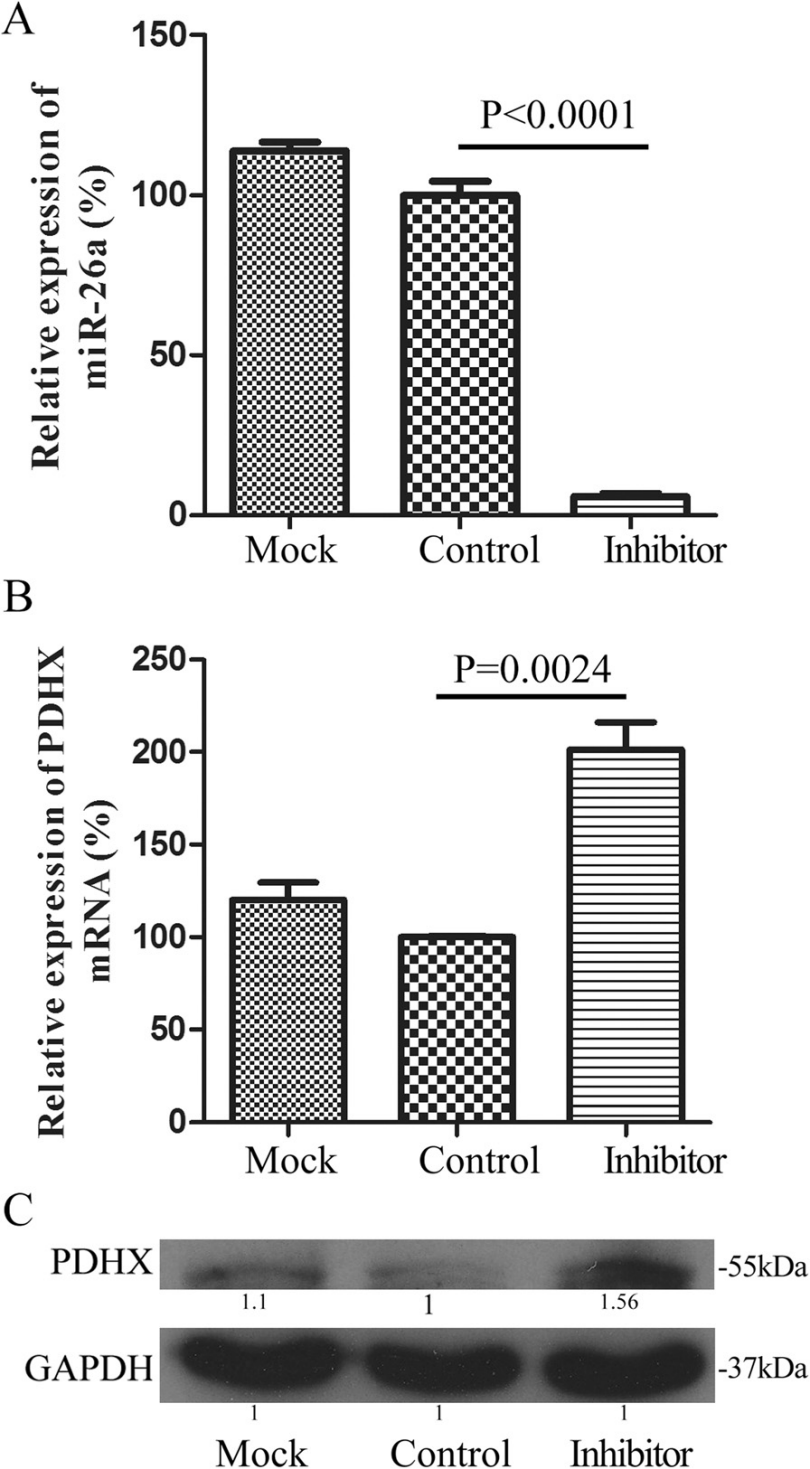
**The correlation of miR-26a expression and its target protein PDHX in SW620 cells.** The expression of PDHX at both mRNA **(B)** and protein levels **(C)** was upregulated in SW620 cells with a decrease of endogenous miR-26a **(A)**. The non-targeting oligonucleotide of miR-26a was used as a control. The untreated SW620 cells were taken as the mock object. The mean values were calculated from three separate experiments. SEM: the standard error of the mean.

Among these CRC cell lines, HCT116 cells were chosen to perform the following studies since it showed the media expression of miR-26a. Firstly, the miR-26a expression plasmid pENTR-miR-26a was transfected into HCT116 cells to detect its influence on the expression level of PDHX. As a result, a marked reduction of PDHX was observed at both mRNA and protein level when miR-26a was overexpressed with almost 2.5-fold (Figure 3A). Compared to the empty vector pENTR-MIRNA, the expression of PDHX mRNA reduced almost 33% (P < 0.001) in HCT116 cells transfected with pENTR-miR-26a (Figure 3B), and PDHX protein was decreased by almost 40% (Figure 3C).Furthermore, the suppression of miR-26a expression was performed in HCT116 cells treated with 30 nM of miR-26a inhibitor, and the random oligonucleotides were taken as negative controls. Compared with the negative control, the mRNA expression of PDHX had a 1.3-fold upregulation (Figure 3E) in HCT116 cells, along with about 70% inhibition of miR-26a (Figure 3D), and the protein expression of PDHX was increased 1.5-fold in the treated HCT116 cells (Figure 3F).We also performed the loss-of function studies in SW620 cells, which have a highly endogenous expression level of miR-26a and a low level of PDHX (Figure 2C-D). Under the condition of a great down-regulation of miR-26a in SW620 cells (Figure 4A, p < 0.01), the mRNA expression of PDHX was significantly increased with 2-fold (Figure 4B, p < 0.01), while the protein level of PDHX was improved to 1.56-fold (Figure 4C). The down-regulation of endogenous miR-26a in SW620 cells could restore PHDX expression, which is similar to the effects with a knock-down of miR-26a in HCT116 cells (Figure 3).

Except to the gain and loss-of function studies, the direct regulatory effect of miR-26a on PDHX expression was also verified by *Renilla* luciferase reporter gene assays. Either the plasmid pwt-PDHX or pmt-PDHX was co-transfected with the miR-26a-expressing pENTR-miR-26a into HEK293T cells respectively. Cells were harvested for assessment of luciferase activity at 48 hours after transfection. Compared with the mutated reporter plasmid pmt-PDHX, when the wild type plasmid pwt-PDHX was co-transfected with the miR-26a-expressing plasmid pENTR-miR-26a, the ectopically expressing miR-26a bound to the 3′ UTR of the wild type plasmid pwt PDHX, then the fused luciferase and the 3′UTR of PDHX was cleaved and subsequently degraded, which resulted in an effective decrease of luciferase activity (Figure 1B). By contrast, in the case of the co-transfection of the mutant plasmid pmt-PDHX with the pENTR-miR-26a, the ectopically expressing miR-26a couldn’t bind to the 3′ UTR of the mutant type plasmid pmt-PDHX, furthermore the luciferase activity did not be decreased. These data showed that miR-26a modulates the PDHX expression by direct targeting the 3′UTR mRNA of PDHX.

### MiR-26a affects cell glucose metabolism

**Figure 5:**
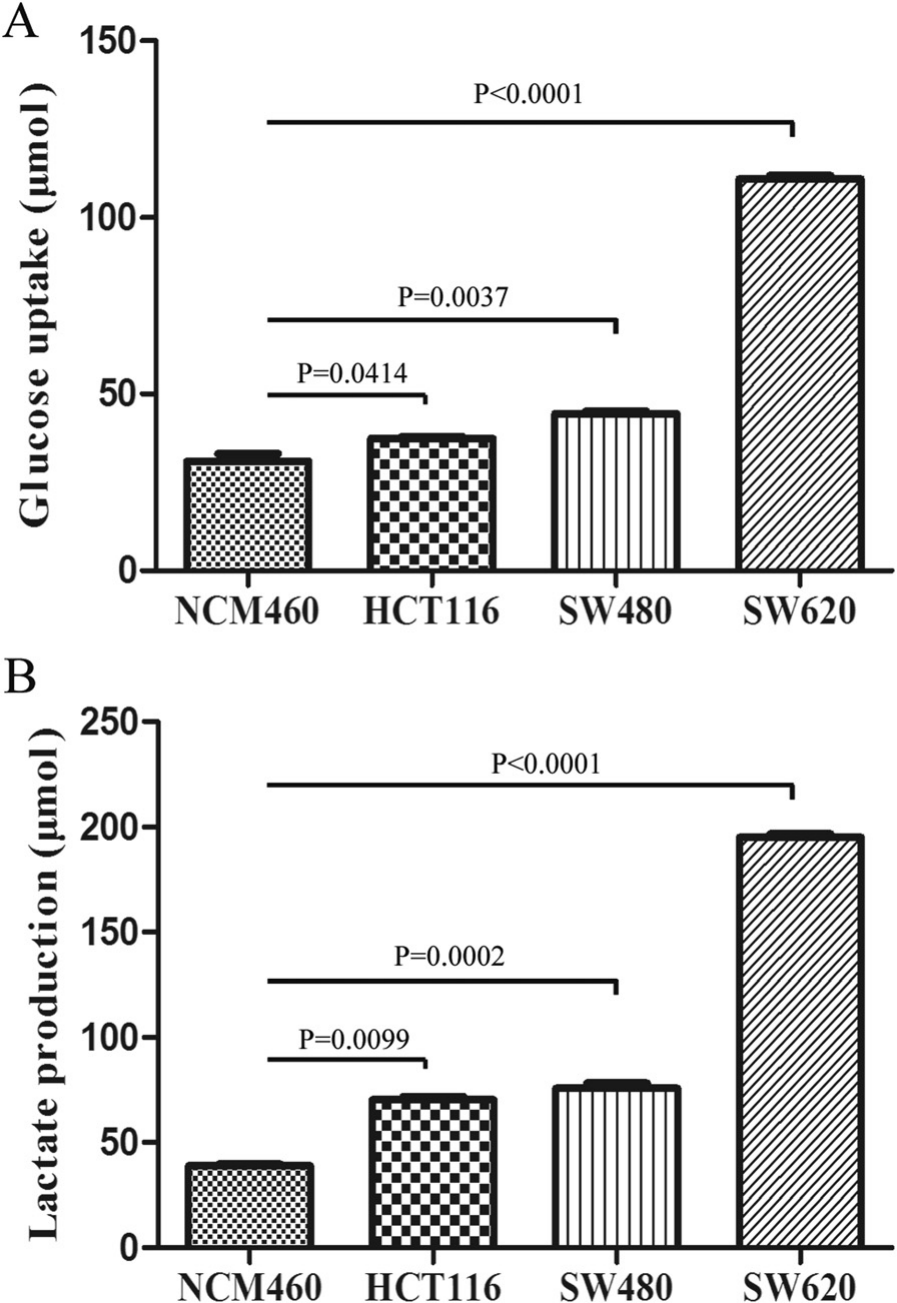
**The glucose consumption (A) and lactate production (B) were measured in colorectal cancer cell lines.** The error bar represented the standard error of the mean (SEM). A Student’s *t*-test was performed to compare the differences between NCM460 and HCT116, SW480 or SW620 cells.

**Figure 6:**
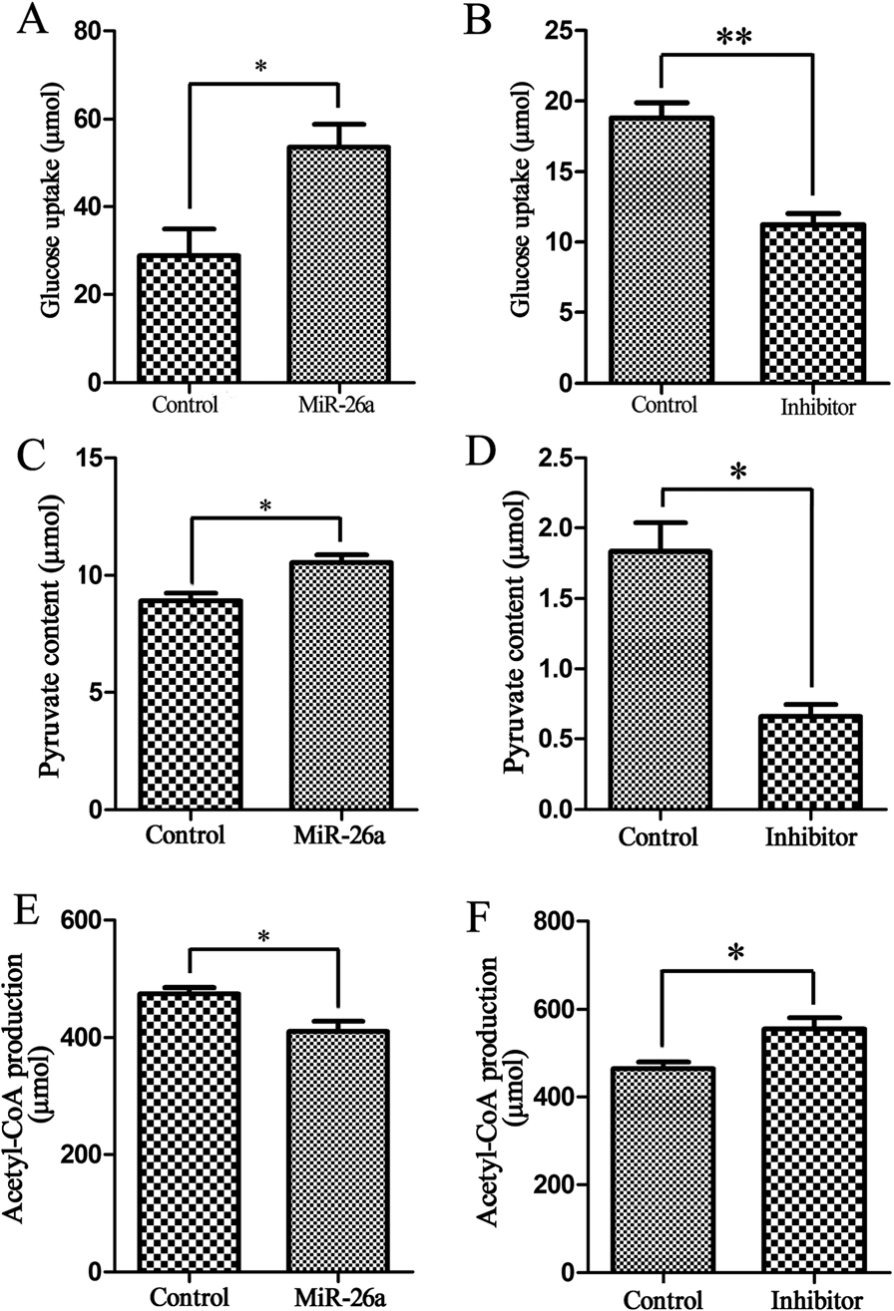
**The miR-26a regulates glucose metabolism in HCT116 cells. (A)** Overexpression of miR-26a, by transfection of pENTR-miR-26a in HCT116 cells, elevated glucose uptake. The empty vector pENTR-MIRNA was used as the control. **(B)** The suppressed expression of miR-26a with its inhibitor treatment greatly induced the decrease of glucose uptake. A non-targeting oligonucleotide was used as the control. The miR-26a inhibits the conversion of pyruvate to acetyl-CoA in HCT116 cells. **(C, D)** The pyruvate content was increased when HCT116 cells transfected with miR-26a-expressing plasmid pENTR-miR-26a, while the acetly-CoA production was decreased. The empty vector pENTR-MIRNA was used as the control. **(E, F)** The suppressed expression of miR-26a with its inhibitor treatment induced the decrease of pyruvate content and the up-regulation of the acetly-CoA production. A non-targeting oligonucleotide was used as the control. The average values ± the standard error of the mean (SEM) of three separate experiments were plotted. *p < 0.05, ** p < 0.005.

The glucose consumption and lactate production were gradually increased among the NCM460, HCT116, SW480 and SW620 cells under physiological conditions (Figure 5). Compared with the normal cell line NCM460, each CRC cell line showed a high level of glucose consumption. The glucose uptake was 30.94, 37.38, 44.42 and 110.99 μmol respectively for NCM460, HCT116, SW480 and SW620 cells. Moreover, there is a positive correlation between the lactate production and the malignant potential of CRC cells. The lactate production was 38.78 μmol, 70.24 μmol, 75.63 μmol and 195.10 μmol from NCM460, HCT116, SW480 to SW620 cells. The lactate content in NCM460 cells was much lower than each of CRC cells. Especially for SW620 cells, it showed more than 5-fold of lactate production than NCM460 cells. In combination with the miR-26a endogenous level in these cells (Figure 2C), it is concluded that the overexpression of miR-26a in CRC cells may cause a preference to glycolysis.In order to investigate miR-26a effects in glucose usage for colon cancer cells, the glucose uptake in HCT116 cells was compared either under the condition of miR-26a overexpression or inhibition. The glucose consumption was increased from 28.86 μmol to 53.58 μmol in miR-26a-overexpressing HCT116 cells by transfecting with pENTR-miR-26a (Figure 6A), showing an almost 2-fold elevation (p < 0.05). Furthermore, we performed a loss-of-function analysis of miR-26a in glucose consumption by using its inhibitor to silence endogenous miR-26a expression. With loss of miR-26a expression, the glucose usage was decreased from 18.82 μmol to 11.25 μmol after being treated with miR-26a inhibitor compared to the control (Figure 6B), indicating a 0.59-fold change (p < 0.005). These evidences strongly demonstrated that the expression level of miR-26a is positively associated with the glucose utilization in colon cancer cells. The overexpression of miR-26a enhanced glucose usage. On the contrary, the inhibition of miR-26a expression would reduce glucose consumption.

**Figure 7:**
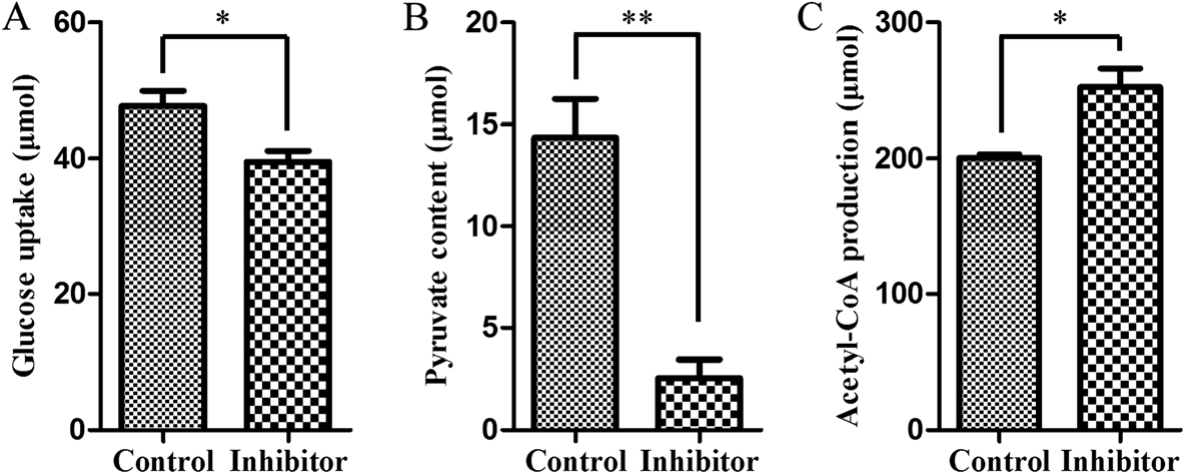
**The miR-26a regulates glucose metabolism in SW620 cells. (A)** The suppressed expression of miR-26a with its inhibitor treatment greatly induced the decrease of glucose uptake. A non-targeting oligonucleotide was used as the control. The miR-26a inhibits the conversion of pyruvate to acetyl-CoA in SW620 cells. **(B, C)** The suppressed expression of miR-26a with its inhibitor treatment induced the decrease of pyruvate content and the up-regulation of the acetly-CoA production. A non-targeting oligonucleotide was used as the control. The average values ± the standard error of the mean (SEM) of three separate experiments were plotted. *p <0.05, **p < 0.005.

**Figure 8:**
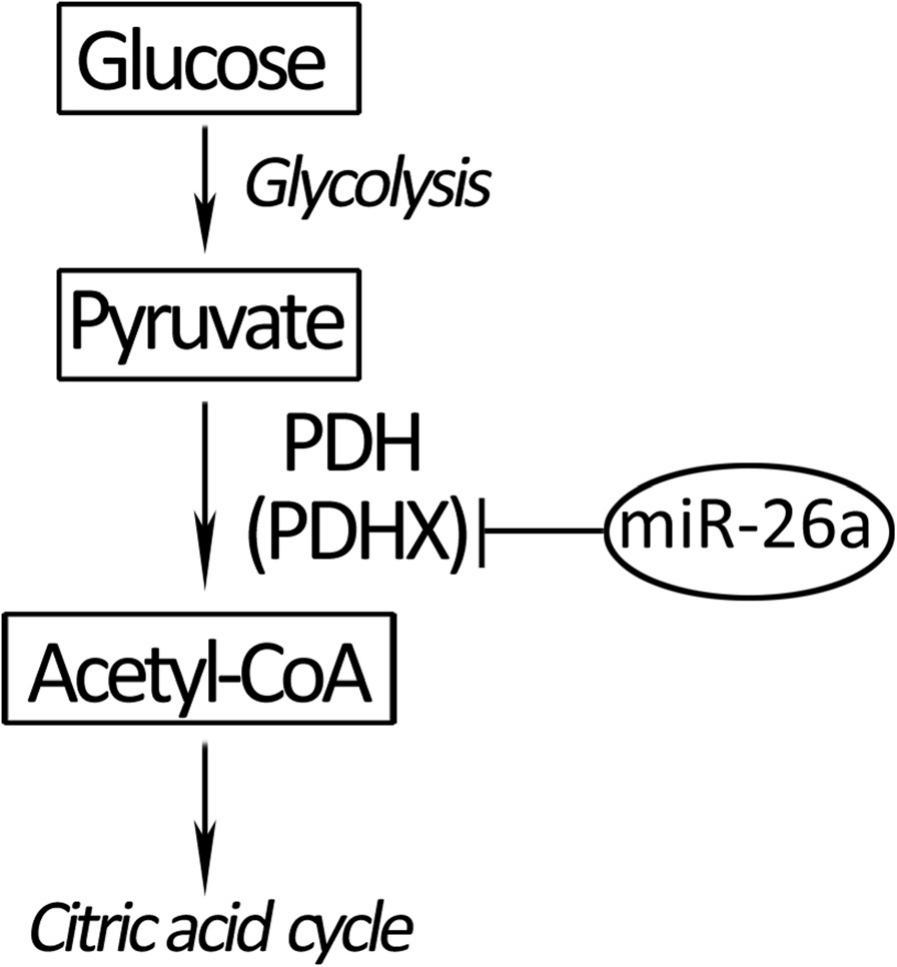
**The molecular mechanism of miR-26a involved in glucose metabolism in CRC cells.** MiR-26a directly targets the PDHX to suppress the conversion of pyruvate to acetyl-CoA, thus blocking the key step of glycolysis to the citric acid cycle. “┤” represents the inhibition effect.

Considering the direct target molecule of miR-26a, we further detected whether the effects of miR-26a on glucose metabolism were mediated its target gene PDHX. PDHX, also known as E3 binding protein (E3BP), is a non-catalytic subunit of PDH complex [18,24] to catalyze the conversion of pyruvate to acetyl-CoA, thereby linking glycolysis to the citric acid cycle [25]. Since PDHX plays a key role in the conversion of pyruvate to acetyl-CoA, we further detected the concentration changes of pyruvate and acetyl-CoA in miR-26a-overexpressing HCT116 cells. As expected, a 1.2-fold increase of pyruvate accumulation (from 8.90 μmol to 10.55 μmol) was detected with the ectopic overexpression of miR-26a (Figure 6C), while the amount of acetyl-CoA (from 474.87 μmol to 410.26 μmol) in HCT116 cells showed a 14% decrease (Figure 6E) (p < 0.05). Besides, the expression inhibition of endogenous miR-26a could induce the decrease of pyruvate content. In detail, the pyruvate content in HCT116 cells was decreased from 1.83 μmol to 0.66 μmol, reducing about 63% (p < 0.05) after being treated with miR-26a inhibitor (Figure 6D). While in the same conditions, the production of acetyl-CoA was effectively increased from 464.87 μmol to 554.90 μmol, raising to almost 1.2 fold (p < 0.05) (Figure 6F).Similarly, we had also performed the loss-of-function studies in SW620 cells to measure the glucose utilization, pyruvate content and acetyl-CoA production. As shown in Figure 7, when the endogenous miR-26a was inhibited, the glucose uptake was decreased from 47.66 μmol to 39.50 μmol and the pyruvate content was decreased from 14.36 μmol to 2.56 μmol, with about 20% (p < 0.05) and 75% (p < 0.01) reduction respectively. While the acetyl-CoA conversion was increased from 200.28 μmol to 252.46 μmol, with almost a 1.3-fold upregulation (p < 0.05). It indicated that the inhibition of miR-26a in SW620 cells could cause similar glucose metabolic effects that were obtained in HCT116 cells.All these results have demonstrated that miR-26a inhibits the conversion of pyruvate to acetyl-CoA though directly targeting the PDHX (Figure 8), which accelerates the glucose consumption to undergo aerobic glycolysis for meeting their increased energy and biosynthesis in colon cancer cells.

## Discussion

The role of miR-26a in carcinogenesis appears to be a complicated one, in the sense that both oncogenic and tumor suppressive effects were reported in cancers. For example, miR-26a enhances lung cancer cell metastasis potential via activating AKT pathway by phosphatase and tensin homolog (PTEN) suppression [26]. Other studies find that miR-26a suppresses cell growth and metastasis through IL-6-Stat3 signaling in hepatocellular carcinoma [27]. Our study showed that miR-26a has an increased level in CRC cells, especially it has much higher expression in high-metastasis SW620 cells (Figure 2C). The finding indicates miR-26a has an oncogenic role in the carcinogenesis of CRC.

More importantly, we also discovered the molecular mechanism of miR-26a involved in glucose metabolism of colon cancer cells. The miR-26a inhibits the expression of PDHX by direct targeting the conserved miR-26a recognition motif of the 3′UTR of PDHX mRNA, which efficiently decreases the process of pyruvate-acetyl-CoA conversion and thus blocks the key step of glycolysis to the citric acid cycle in glucose metabolism. The PDHX is a non-catalytic subunit of the PDH complex, which is located in the mitochondrial matrix and is central to mitochondrial fuel metabolism [28]. The PDH complex catalyzes the irreversible oxidation of pyruvate to acetyl CoA, a rate-limiting step under aerobic conditions for the oxidative removal of glucose and pyruvate and for other 3-carbon metabolites (alanine and lactate) in equilibrium with pyruvate [29]. Except to the non-catalytic subunit PDHX, the PDH complex contains three catalytic subunits (pyruvate dehydrogenase E1, dihydrolipoamide transacetylase E2 and dihydrolipoamide dehydrogenase E3) and two regulatory subunits (E1 kinase and E1 phosphatase). It is known that PDHX is required for tethering E3 dimers to the E2 core and this specific binding is essential for a functional PDH complex. Our results provided experimental evidences to verify the bioinformatics speculation that miR-26a has a high possibility to regulate PDHX in lung cancer [3]. So far, it is the first report to explore the miR-26a-regulated glucose metabolism via targeting PDHX in CRC, which is helpful for understanding miRNA functions in the biochemical processes of the Warburg effect in tumors.

Glucose utilization was changed significantly in HCT116 cells when miR-26a was ectopically overexpressed or inhibited. It seems that miR-26a might prompt glucose uptake to meet the need of energy and biosynthesis in cancer cells. Most mammalian cells import glucose by a process of facilitative diffusion mediated by members of GLUTs family [30]. The effects of miR-26a on the expression and/or the translocation of the GLUT proteins could be very interesting to further investigate in our following research. Furthermore, growing evidence has shown that cancer cell metabolism is also controlled by external responses to the tumour microenvironment [9]. For example, cancer-associated fibroblasts have been reported to undergo Warburg metabolism and mitochondrial oxidative stress [31]. They are possibly correlated with metabolic reprogramming of cancer cells toward Warburg metabolism by rapidly expressing carbonic anhydrase IX, which could result in extracellular acidification [32]. These factors will be considered in our further study.

Recently, microRNA molecules are already entering the clinic as potential diagnostic and prognostic biomarkers, as well as therapeutic targets or agents [33]. The miRNAs as important regulators of metabolism have garnered much interest not only from a scientific point of view but also from a clinical perspective [1]. The function of miRNAs on cellular metabolism reveals molecular strategies for controlling metabolic flux by miRNAs in living organisms, thus lighting up one aspect of miRNA therapeutics. The use of miRNAs, such as oligonucleotide complementary [34] or antisense oligonucleotides [35] in miRNA inhibition, to suppress cell metabolism altering will hopefully lead to a new therapeutic strategy for malignant cancer [36,37]. A high rate of glycolysis to obtain energy is a commonly observed feature in cancers, and previous studies usually focus on miRNA roles in targeting the key enzymes of glycolysis, including hexokinase-2 [3], and pyruvate dehydrogenase kinase [38,39]. Our data indicated that miR-26a also regulates the process of pyruvate-acetyl-CoA conversion which is the former reaction of entering the citric acid cycle. Previous studies have shown that miR-26a is significantly associated with disease progression and therapy response in CRC [39-42]. Therefore, the miR-26a is a potential cancer therapeutic target by regulating glucose metabolism in CRC, which also provides a promising anti-neoplasia strategy.

## Conclusions

The expression levels of miR-26a are associated with the malignant degrees of CRC cells. MiR-26a has key roles in regulating glucose metabolism by direct targeting PDHX in CRC cells , which inhibits the conversion of pyruvate to acetyl CoA in tricarboxylic acid cycle. These novel findings are helpful for understanding the CRC development and indicate a promising anti-neoplasia strategy.

## Abbreviations

miR-26a: microRNA-26a; GLUT: Glucose transporter; UTR: Untranslated region; PDHX: Pyruvate dehydrogenase protein X component; miRNAs: microRNAs; CRC: Colorectal cancer; acetyl-CoA: acetyl coenzyme A.

## Competing interests

The authors declare that they have no competing interests.

## Authors’ contributions

Chen B performed most of the experiments and wrote the paper; Liu J performed bioinformatics analysis; Jin X, Liu Y and Lu W co-worked with Xia Z and Yuan Q to perform cellular experiments. Zhao X and Xu N gave suggestions on this project. Liang S conceived, instructed all experiments and revised the paper. All authors read and approved the final manuscript.

## Pre-publication history

The pre-publication history for this paper can be accessed here:

http://www.biomedcentral.com/1471-2407/14/443/prepub

## Supplementary Material

Additional file 1: Table S1 The nucleotide sequences cloned in pENTR-miR-26a. Click here for file

Additional file 2: Table S2 The full length nucleotide sequences of PDHX 3′UTR cloned in the plasmid pwt-PDHX and pmt-PDHX. Click here for file
