# Supplementary material for: MicroRNA-26a regulates glucose metabolism by direct targeting PDHX in colorectal cancer cells
Source: BMC Cancer. 2014 Jun 16;14:443. doi: 10.1186/1471-2407-14-443 (PMC4071217; doi:10.1186/1471-2407-14-443)
Supplement: Additional file 1: Table S1 — The nucleotide sequences cloned in pENTR-miR-26a. [file 1471-2407-14-443-S1.doc]

Additional file 1: Table S1. The nucleotide sequences cloned in pENTR-miR-26a.

| CGTGACTGTAAGCATGACTGGCCTGCCCACTGGTGACCCATTCTTGCAGGAGCTCCCCCACCCTCCATCCTGGCTGTGCTGTGATATCACAAGGTCCCAGGGCTGGGGTCAGAAATTCTCTCCCGAGGGAATGAAGCCACAGGAGCCAAGAGCAGGAGGACCAAGGCCCTGGCGAAGGCCGTGGCCTCGTTCAAGTAATCCAGGATAGGCTGTGCAGGTCCCAATGGGCCTATTCTTGGTTACTTGCACGGGGACGCGGGCCTGGACGCCGGCATCCGGGCTCAGGACCCCCCTCTCTGCCAGAGGCACCAACACCAGAGTTCACAAATCAGTCTCCTGCCCTTTGCATGTAGCAAAGCTTGCTTTCCAAGCAGGACTCCCAGGGTGACTGAGGACTACAGGCCACACCCGTTTGCTGGCAACGAGGAGTCTTGTGGGCAAAGTGGGAGTCTGCAGCTGTT |
| --- |

The 461bp fragments were included in pENTR-miR-26a. The inserted nucleotide sequences of pre-miR-26a were underlined.
