# Supplementary material for: MicroRNA-26a regulates glucose metabolism by direct targeting PDHX in colorectal cancer cells
Source: BMC Cancer. 2014 Jun 16;14:443. doi: 10.1186/1471-2407-14-443 (PMC4071217; doi:10.1186/1471-2407-14-443)
Supplement: Additional file 2: Table S2 — The full length nucleotide sequences of PDHX 3′UTR cloned in the plasmid pwt-PDHX and pmt-PDHX. [file 1471-2407-14-443-S2.doc]

Additional file 2: Table S2. The full length nucleotide sequences of PDHX 3’UTR cloned in the plasmid pwt-PDHX and pmt-PDHX.

| 1. The nucleotide sequences of PDHX 3’UTR cloned in the plasmid pwt-PDHX : |
| --- |
| TCCTCAAAGATAAGAAGTTGGTGTTCAGCTTAGTTGATTCAGTAGTTGTTACCAAGAAACATATGTTATAGGAAAACAACTTGGTATTTAAGTATGAAGTGGATGAAATGTTTATTTATTTAAGGTGAAAGCATTTGACCCAGGGTGTCTTCATCTTCAATTTGGGTTTAATGTTATAGAAATAAATGATGATAAACTCTAACTAATAAAGGAAAGAGAATATTTGGTTACTCAGATCCATTTTTAACCTCTGGTGCTGTATAAAGGGAATATTAAACTAGATGTAAATCAAAGTATATGTTTGGCTCATTTGAGCATTTTGGAATATTTGAGAATGTATGATACATGTAAAATTAAAAAAACTATTAGAACTGTACCATAATTATGTTGAAGGTAGAAGTGATCTTCAAAGAGATGGCCATTAACTTAGCAGTGGGACCTCACTTTTACAAGCACTGCTCTAGATA**TACTTGAA**GAATTTAATAGGTACAGAAGTTTATTCTGGATAATAAATAAATAAGGATCACACTGTATTAGGGGTTATGGCAACATTATTGAATTTTTTATGTACATAAAGCCATATGTTTAGGGTGGTTTCTATCTGTCTTGTTTTTCACTTATATAACACTGTGAACTTCTAAAGCAAGAGGATAAAAGAAGCATGAATGAAAAGAATGACATTTCAAAAAAATGGTTCAATGAAAAACTATAGCTAAAATATGTAAACCTTTCTAGGTAAACCGCTTGCCTTCATCTTGAGTCGGAATATATTTAAATAAATTGTGTTATCTCTTGCC  The eight nucleotides corresponding to miR-26a seed region were underlined. |
| 2. The nucleotide sequences of the 3’UTR cloned in the plasmid pmt-PDHX: |
| TCCTCAAAGATAAGAAGTTGGTGTTCAGCTTAGTTGATTCAGTAGTTGTTACCAAGAAACATATGTTATAGGAAAACAACTTGGTATTTAAGTATGAAGTGGATGAAATGTTTATTTATTTAAGGTGAAAGCATTTGACCCAGGGTGTCTTCATCTTCAATTTGGGTTTAATGTTATAGAAATAAATGATGATAAACTCTAACTAATAAAGGAAAGAGAATATTTGGTTACTCAGATCCATTTTTAACCTCTGGTGCTGTATAAAGGGAATATTAAACTAGATGTAAATCAAAGTATATGTTTGGCTCATTTGAGCATTTTGGAATATTTGAGAATGTATGATACATGTAAAATTAAAAAAACTATTAGAACTGTACCATAATTATGTTGAAGGTAGAAGTGATCTTCAAAGAGATGGCCATTAACTTAGCAGTGGGACCTCACTTTTACAAGCACTGCTCTAGATA**TCACCAAT**GAATTTAATAGGTACAGAAGTTTATTCTGGATAATAAATAAATAAGGATCACACTGTATTAGGGGTTATGGCAACATTATTGAATTTTTTATGTACATAAAGCCATATGTTTAGGGTGGTTTCTATCTGTCTTGTTTTTCACTTATATAACACTGTGAACTTCTAAAGCAAGAGGATAAAAGAAGCATGAATGAAAAGAATGACATTTCAAAAAAATGGTTCAATGAAAAACTATAGCTAAAATATGTAAACCTTTCTAGGTAAACCGCTTGCCTTCATCTTGAGTCGGAATATATTTAAATAAATTGTGTTATCTCTTGCC  The eight nucleotides corresponding to miR-26a seed region were randomly mutated (underlined). |
